# Automatic flow system for simultaneous determination of iron and chromium in steel alloys employing photometers based on LEDs as radiation source

**DOI:** 10.1155/S1463924603000014

**Published:** 2003

**Authors:** Ridvan N. Fernandes, Boaventura F. Reis, Luís Fernando P. Campos

**Affiliations:** 1 Departamento de Química Universidade Federal do Maranhão Brazil; 2 Centro de Energia Nuclear na Agricultura Universidade de São Paulo Av. Centenário 303, Box 96 Piracicaba SP 13400-970 Brazil

## Abstract

A multicommutated flow system for simultaneous determination of iron and chromium in steel alloys by photometry is described. The flow network consisted of an automatic injector and four solenoid valves assembled to form two independent analytical pathways, each one comprising reaction coils and a flow cell. The light source (LED) and detector (photodiode) were attached to the flow cells to form a compact unit. The flow system was microcomputer controlled by Quick BASIC 4.5 software, which carried out all steps of the analytical procedure. The feasibility of the system was proved by the determination of iron and chromium in steel alloys and its accuracy was accessed by comparing results with those obtained by plasma atomic emission spectrometry (ICP-AES). No significant difference at the 95% confidence level was observed. Other profitable features such as low reagent consumption (0.33 mg 1,10-phenantroline and 0.03 mg 1,5-diphenylcarbazide per determination); relative standard deviations (n = 5) of 0.4% for iron and 1.2% for chromium; and an analytical throughput of 160 determinations per h were also achieved.


Journal of Automated Methods & Management in Chemistry
Vol. 25, No. 1 (January–February 2003) pp. 1–5

Taylor & Francis

Taylor & Francis Group

### Automatic flow system for simultaneous determination of iron and chromium in steel alloys employing photometers based on LEDs as radiation source

**Ridvan N. Fernandes[1], Boaventura F. Reis[2]* and Luís Fernando P. Campos[2]**

[1]*Departamento de Química, Universidade Federal do Maranhão, Brazil*
[2]*Centro de Energia Nuclear na Agricultura, Universidade de São Paulo, Av. Centenário, 303, Box 96, 13400-970, Piracicaba, SP, Brazil*

*A multicommutated flow system for simultaneous determination of iron and chromium in steel alloys by photometry is described. The flow network consisted of an automatic injector and four solenoid valves assembled to form two independent analytical pathways, each one comprising reaction coils and a flow cell. The light source (LED) and detector (photodiode) were attached to the flow cells to form a compact unit. The flow system was microcomputer controlled by Quick BASIC 4.5 software, which carried out all steps of the analytical procedure. The feasibility of the system was proved by the determination of iron and chromium in steel alloys and its accuracy was accessed by comparing results with those obtained by plasma atomic emission spectrometry (ICP-AES). No significant difference at the 95% confidence level was observed. Other profitable features such as low reagent consumption (0.33 mg 1,10-phenantroline and 0.03 mg 1,5-diphenylcarbazide per determination); relative standard deviations ($n = 5$) of 0.4% for iron and 1.2% for chromium; and an analytical throughput of 160 determinations per h were also achieved.*

### Introduction

The simultaneous determination of two or more analytes at a time by flow-injection analysis became very attractive after the work proposed by Stewart and Ruzicka (1976) [1]. Afterwards, a large number of flow procedures for multiparameter determination per sample using different detection techniques have been described [2–6].

When a multidetermination flow system is implemented using UV-Vis spectrophotometry as the detection technique, the reagents' incompatibility is one difficulty that may appear. This drawback has been surmounted by designing flow systems based on merging zones [2, 3] or on sandwich-technique approaches [4]. When analytes compound absorb radiation at the same wavelengths, the flow networks have been designed to determine each analyte at a different time [5]. On the other hand, if chemical species absorb at a different wavelength, simultaneous determination had been carried out, nevertheless equipment with the ability to sweep automatically the wavelengths have been employed [6, 7].

A light-emitting diode (LED) has been employed as a radiation source in some photometric procedures, its advantages being robustness and low current consumption [8, 9]. Nevertheless, depending on the LED type, the width of the emission band can range from 30 to 100 nm [10–13]. However, by carefully selecting the methods, LEDs can became a good option as a radiation source in flow system when multidetermination is performed with photometric detection employing non-expensive instrumentation [14–17].

The flow network for multicomponent determination can became complex, mainly when the selected spectrophotometric methods required two reagent solutions per analyte [3, 18]. This difficulty can be minimized by employing the multicommutation approach that allowed facilities to handle several reagent solutions using a single pumping channel [19, 20].

In the present work, the intention is to develop a photometric flow set-up for the determination of two analytes at the same time using LEDs as the radiation source and a photodiode as the detector. The flow network was designed based on the multicommutation approach [21, 22], which aimed to implement a compact and inexpensive flow system for simultaneous determination of iron and chromium in steel alloys, also presenting a low reagent consumption, which is an inherent feature of the multicommutated flow system [22, 23]. As chromogenic reagents, 1,10-phenantroline and 1,5-diphenylcarbazide were selected for iron and chromium, respectively.

### Experimental

*Reagents, standards and samples*

All solutions were prepared with analytical-grade reagents, and freshly distilled and deionized water was used throughout.

A 0.06% (w/v) 1,5-diphenylcarbazide solution was prepared by dissolving 0.06 g in 2 ml 96% (v/v) ethanol and making the volume up to 100 ml with water. This solution, which was stored in refrigerator, could be use for at least 1 week. Before use, a 20-ml aliquot was equilibrated to laboratory temperature.

A 0.25% (w/v) 1,10-phenantroline solution was prepared by dissolving 0.5 g in 100 ml of hot water (≅70°C). After cooling to room temperature, the volume was made up to 200 ml with water. This solution was stable by 1 week.

* To whom correspondence should be addressed. e-mail: reis@cena.usp.br

A 0.5 $mol\,l^{-1}$ hexamine buffer solution, pH 4.9, was prepared by adjusting the pH with HCl.

A 1.0% (w/v) ascorbic acid solution was prepared by dissolving 0.5 g in 50 ml on the examine buffer solution. This solution was prepared every day.

A 1000 $mg\,l^{-1}$ iron(III) stock solution was prepared by dissolving 1.0 g metallic iron in 10 ml concentrated HCl plus 10 ml $HNO_3$ concentrated. After dissolution, the volume was made up to 1000 ml with water. Working standard solutions 0, 5, 15, 30, 60, 90 and 120 $mg\,l^{-1}$ $Fe^{3+}$ in 0.5 $mol\,l^{-1}$ HCl medium were prepared by appropriated dilution from the stock solution.

A 1000 $mg\,l^{-1}$ chromium(VI) stock solution was prepared by dissolving 3.7535 g potassium dichromate in 1000 ml water. Working standard solutions 0, 5, 15, 30, 45, 60 and 75 $mg\,l^{-1}$ $Cr^{6+}$ in 0.5 $mol\,l^{-1}$ HCl medium were prepared by appropriated dilution from the stock solution.

Sample solutions for iron and chromium determination were prepared as described by elsewhere [5].

*Apparatus*

The equipment set-up consisted of two LEDs-based photometers constructed to implement the work; an IPC8 Ismatec peristaltic pump furnished with Tygon tubes; a home-made automatic commutator injector with two commutation sections, which was controlled by means of two solenoids attached to its sliding bar [21]; four 161T031 three-way solenoid valves (NResearch, Stow, MA, USA); a 486 microcomputer equipped with a PCL711S interface card (American Advantech, San Jose, CA, USA); and a home-made electronic interface to match voltage and current intensities required to switch on the commutator injector and solenoid valves [18]. Reaction coils and flow lines were of polyethylene tubing (i.d. = 0.8 mm).

The flow system was controlled by the microcomputer running a software written in QuickBasic 4.5, which was designed to carry out all steps involved in the proposed analytical procedures comprising solutions handling and data acquisition.

*Detection system*

A block diagram of the LEDs-based detectors is shown in figure 1. The two LEDs ($L_1$, $L_2$) employed as the radiation source presented a maximum wavelength at 560 nm (half wide ≈50 nm). The maximum absorption of the compounds of iron and chromium occurred at 520 and 536 nm, respectively, thus presenting a good overlap with the emitted radiation band.

The photodetectors ($Det_1$, $Det_2$) were based on the photodiode supplied by RS Data Library (Catalogue No. RS 308-067). Each detection set-up had an LED, flow cells ($F_1$, $F_2$) with 10 mm optical length and 100 µl inner volume, and a photodiode. These devices were assembled in a black acrylic block forming compact units. Both photometers presented similar physical structures and the electronic diagram is shown in figure 2. The operational amplifier (741) was assembled to work with unitary gain in order to provide the impedance marriage and to allow the baseline adjusting. The analytical signal was the potential difference between pin 2 and the 8.2 V set to pin 1 as reference, which was supplied by the Zener diode in series with the 3.3 Ωk resistor. The reference potential (8.2 V) was also used by means of the variable resistor (R) to adjust the value of the baseline measurement. The LED emission intensity was adjusted by controlling the current intensity applied to the base of the transistor (BC547). High radiation intensity can saturate the detector, thus causing a hindering of its response, by other hand, an excessive reduction of the radiation intensity decreased its linear response range.

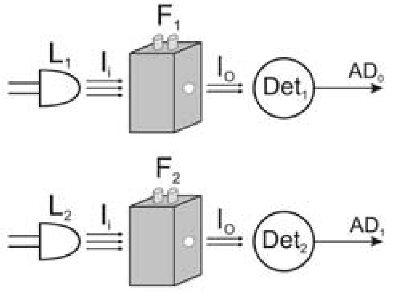

*Figure 1. Bloc diagram of the photometers. $L_1$, $L_2$, light-emitting diode (LED), $\lambda = 560$ nm; $F_1$, $F_2$, flow cell, 10 mm path length, 100 µl inner volume; $Det_1$, $Det_2$, phototransistor; $AD_0$, $AD_1$, input of the analogue/digital interface card; Ii, Io, radiation intensity.*

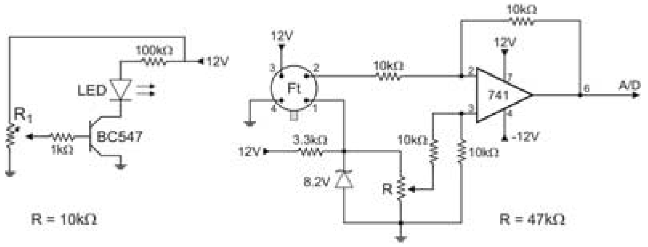

*Figure 2. Electronic diagram of the photometer. Ft, photodetector; Catalogue No. RS 308-067.*

The signals generated by the photometers were read by the microcomputer through the analogue input of the PCL711S interface card. This task was done by coupling the output of the operational amplifier (741) of the photometers ($F_1$, $F_2$) to the $A_0$ and $A_1$ analogue inputs of the interface card, which were selected by software using the interface analogue multiplex. The control software was developed to carry out the handling of the sample and reagents solutions and to perform data acquisition as indicated on its flow chart shown in figure 3.

*Flow diagram and experimental variables*

The flow network was designed to implement the multicommutation and binary sampling approaches and the flow diagram is shown in figure 4. When the software was run, it request the actual values of the system control variables summarized in table 1. Afterwards, all the steps in the analytical process were carried out without any operator assistance.

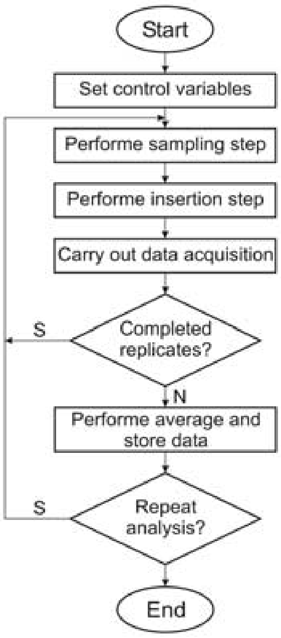

*Figure 3. Flow chart of the software.*

Initially, all solenoid valves were switched off (figure 4) and the carrier solutions ($C_1$, $C_2$) were flowing by aspiration through the valves ($V_3$, $V_4$) and reaction coils ($B_2$, $B_3$) towards the detectors ($Det_1$, $Det_2$). The software was designed to work following the sequence depicted in the valves timing course of figure 1, i.e. the basic strategy of the binary sampling concept [19–23]. To begin the analytical process, the microcomputer sent through the PCL711 interface card a control signal to displace the injector-sliding bar to the sampling position (figure 4). This was done by switch on during a time interval ($d_i = 1$ s) one of the solenoids attached to the injector sliding bar [21]. Afterwards, valves $V_1$ and $V_2$ were switched on/off several times as indicated in the valves' timing course. This was done to maintain the time intervals as defined in table 1. When valve $V_2$ was switched on, the carrier solution stream ($C_2$) was halted and the sample solution (S) flowed through this valve and coil $B_1$ towards the sampling loop $L_2$. When valve $V_1$ was switched on, the stream of solution sample (S) was halted and the reagent solution ($R_1$) flowed through this valve towards the sampling loop $L_1$. When valves $V_1$ and $V_2$ were switched off, the initial solutions flowed again. Henceforth, an on/off valve switching will be referred as a sampling cycle. A sampling cycle was repeated several times to fill the sampling loops. Under this condition, sampling loops $L_1$ and $L_2$ were loaded with strings comprising sample slugs in tandem with slugs of reagent solution $R_1$ and carrier solution $C_2$, respectively. After the sampling step had been completed, the injector sliding bar was displaced to the injection position (hatched surface) by powering the other solenoid attached to injector sliding bar [21]. Afterwards, the solenoid valves $V_3$ and $V_4$ were switched on/off several times (table 1) to insert into the reaction coils $B_2$ and $B_3$ a sequence of sample slugs in tandem with slugs of the reagent solutions $R_2$ and $R_3$.

Mixing of the solutions occurred while the sample zones were displaced by the carrier solutions towards the detectors $Det_1$ and $Det_2$. The signals related to chromium and iron concentrations were read by a mean of the PEL711s interface card coupled to the detector outputs as a time function and stored for further treatment to determine the concentration of the analytes. While this task was in progress, the data were also displayed on the

*Table 1. System control variables.*

| Step | Valve $V_1$ | Valve $V_2$ | Valve $V_3$ | Valve $V_4$ | Cycle | Time duration (s) |
|---|---|---|---|---|---|---|
| Sampling/Cr | 0 | I | 0 | 0 | 4 | 0.2 |
| | 0 | 0 | 0 | 0 | | 0.2 |
| Sampling/Fe | I | 0 | 0 | 0 | 10 | 0.2 |
| | 0 | 0 | 0 | 0 | | 0.2 |
| Insertion/Cr | 0 | 0 | I | 0 | 10* | 0.2 |
| | 0 | 0 | 0 | 0 | | 0.2 |
| Insertion/Fe | 0 | 0 | 0 | I | 20* | 0.2 |
| | 0 | 0 | 0 | 0 | | 0.2 |
| Data acquisition | 0 | 0 | 0 | 0 | | 25 |

Symbols I and 0 indicate valves switched on and off, respectively.
* Number of reagents' slugs inserted in the sample zones.

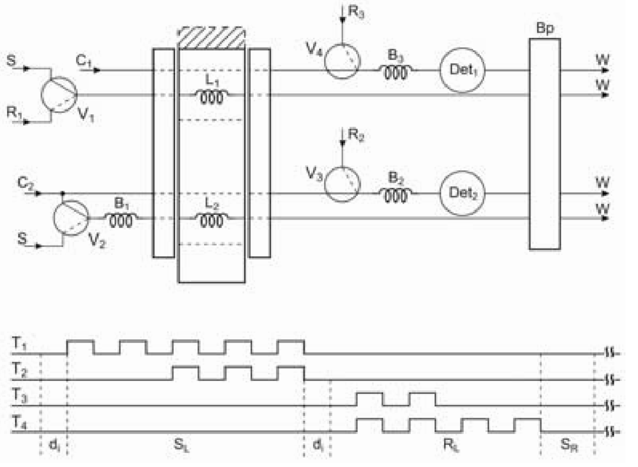

*Figure 4. Flow diagram of the system. The three-rectangular surface is an overview of the injector. The hatched area is the alternative position of the sliding bar (central part) and the dashed lines are inner holes; $V_1$, $V_2$, $V_3$, $V_4$, three-way solenoid valves, solid lines into the valves symbols were the fluid pathway, while valves were off and dashed lines were the alternative pathway when valves were switched on; $L_1$, $L_2$, sampling loops, 25 and 8 cm long, respectively; $B_1$, dilution coil, 25 cm long; $B_2$, $B_3$, reaction coils, 100 cm long; $Det_1$, $Det_2$, photometers; Bp, peristaltic pump; $C_1$, carrier solution for iron determination, 0.5 mol $l^{-1}$ hexamine buffer solution at pH 4.9; $C_2$, carrier solution for chromium determination, 0.5 mol $l^{-1}$ HCl; W, waste; $R_1$, 1.0% ascorbic acid solution; $R_2$, 0.06% (w/v) 1,5-diphenylcarbazide solution; $R_3$, 0.25% (w/v) 1,10-phenantroline solution. $T_1$, $T_2$, $T_3$, $T_4$ = valves $V_1$, $V_2$, $V_3$, $V_4$ timing course; d, injector displacement; $S_L$, sample loading step; $R_l$, reagents' loading step; $S_R$, signal reading step. Sampling loops, coils and flow lines were of polyethylene tubing, 0.8 mm i.d. The high level of the timing course line indicates that the related valve was switched on.*

microcomputer screen while the analytical process was run. The data acquisitions were carried out by sharing the analogue/digital converter of the PCL711 interface card, which afford facilities to read up to eight analogue signals sequentially. Afterwards, the sliding bar of the injector was displaced to the initial position to begin the next analytical run.

The experimental variables such as the pumping flow rates, the time intervals to switch the solenoid valves on/off, the sampling cycle number to load the sampling loops, the time interval to read the analytical signals (table 1) were settled before the start of the experiment. After the experimental variables had been established, iron and chromium were simultaneously determined in a set of steel alloy samples.

### Results and discussion

As depicted in the valve timing regime in figure 4, during the sampling step for chromium determination, the solenoid valve $V_2$ underwent an on/off switching sequence. As indicated in table 1, these time intervals were both fixed at 0.2 s. The flow rate was maintained at 33.3 $\mu l\,s^{-1}$, thus when a sampling cycle was carried out (one on/off valve switching), a sample slug of 6.6 µl was inserted into the dilution coil $B_1$, and afterwards a carrier solution slug with equal volume was inserted while valve $V_2$ was maintained off. Taking into account the concentration range of chromium, the sampling loop $L_2$ and dilution coil $B_1$ were settled at 8 and 25 cm (40 and 125 µl), respectively. To assure the appropriated dilution, four sampling cycles were carried out. Under this condition, a sample solution underwent a dilution >50%, which was required to match the sample concentration with the linear response range of the photometer. The reagent solution ($R_2$) was added to the sample zone by switching valve $V_3$ on/off 10 times. As in the sampling step, the time intervals (on/off) were both fixed at 0.2 s, therefore a volume of 66 µl 1,5-diphenylcarbazide was used per determination.

For iron determination, the length of sampling loop $L_1$ was fixed at 25 cm (125 µl) and the time interval to switch valve $V_1$ on/off was settled at 0.2 s. The flow rate was maintained at 33.3 $\mu l\,s^{-1}$. Thus, to fill the sampling loop $L_1$, 10 sampling cycles were carried out. Under this condition, the reaction to reduce $Fe^{3+}$ ions to $Fe^{2+}$ occurred during the sampling step. The 1,10-phenantroline solution ($R_3$) was added to the sample zone by switching valve $V_4$ (on/off) 20 times, thus inserting a solution volume of 132 µl.

As can be seen in the flow diagrams (figure 4), the two systems were assembled employing the same injector, nevertheless the flow pathways were completely independent allowing simultaneous solutions handling for both analytes. The control software was designed to read the signals generated by the photometers $Det_1$ and $Det_2$ sequentially. The analogue-to-digital converter of the PCL771S interface card presented a converting time of 25 µs. Thus, when considering this feature, the software was designed to read each photometer continuously for 200 times. In this sense, each datum stored and displayed

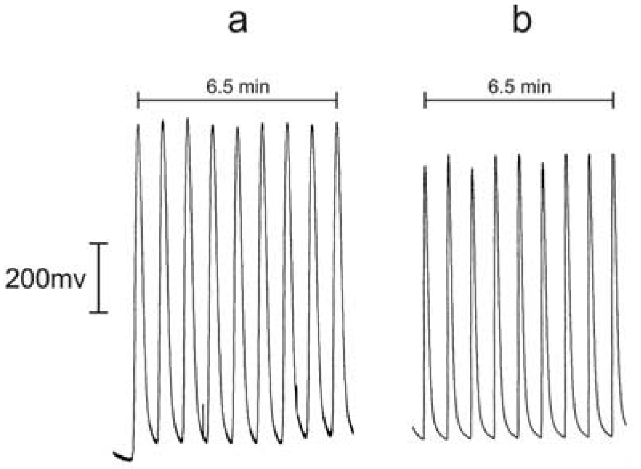

*Figure 5. Recorder tracing. The sets (a) and (b) refer to iron (100 mg $l^{-1}$) and chromium (100 mg $l^{-1}$) determination, respectively.*

on the computer screen (figure 5) was the average of 200 sequential readings of each photometer. Considering other computer tasks related to data acquisition, such as average calculation and datum save, the time interval spent was <50 ms. In this sense, each peak profile shown in figure 5 was plotted using at least 200 measurements.

Both photometers presented good stability (figure 5) characterized by relative standard deviations (RSD) of 0.4% for iron and 1.2% for chromium. Apart from these recorders, one can deduce that an analytical throughput of 160 determinations per h was achieved.

The feasibility of the system was ascertained by processing a set of steel alloy solutions yielding the results shown in table 2. Accuracy was assessed by comparing the results with those obtained with induced coupled argon plasma atomic emission spectrometry (ICP-AES), and no significant difference at the 95% confidence level was observed. Others profitable features—such as linear response, which ranged from 5.0 to 75.0 mg $l^{-1}$ for chromium ($R = 0.997$) and from 5.0 to 120.0 mg $l^{-1}$ for iron ($R = 0.998$); and low reagent consumption, 32 and 330 µg per determination for chromium and for iron, respectively—were also achieved.

*Table 2. Comparison of results.*

| Sample | Iron (%) | | Chromium (%) | |
|---|---|---|---|---|
| | Proposed System | ICP-AES | Proposed System | ICP-AES |
| 1 | 28.62 ± 0.42 | 28.70 ± 0.03 | 32.86 ± 0.37 | 31.90 ± 0.07 |
| 2 | 16.38 ± 0.10 | 15.90 ± 0.08 | 57.82 ± 0.53 | 56.70 ± 0.09 |
| 3 | 29.38 ± 0.74 | 28.90 ± 0.01 | 61.28 ± 0.63 | 58.01 ± 0.01 |
| 4 | 36.82 ± 0.27 | 35.90 ± 0.08 | 46.66 ± 0.45 | 46.40 ± 0.26 |
| 5 | 63.07 ± 0.60 | 62.00 ± 0.12 | 14.41 ± 0.23 | 15.90 ± 0.06 |

Results are the average of three sequential measurements.

### Conclusions

The system is very simple to build and easy to use. The control software carried out all steps of the analytical procedure following the set of parameters previously decided upon (table 1). Considering the following parameters, the results were comparable with those obtained by ICP-AES: a high throughput capability, a low reagent consumption, a linear response range for the photometers and robustness, and it can be concluded that the system is appropriated for use in routine analysis laboratory.

### Acknowledgements

The authors are grateful to CAPES, FAPESP, CNPq and FINEP/PRONEX.

### References

1. Stewart, J. W. B. and Ruzieka, J., *Anal. Chim. Acta*, **82** (1976), 137.
2. Bergamin F°., H., Zagatto, E. A. G., Krug, F. J. and Reis, B. F., *Anal. Chim. Acta*, **101** (1978), 17.
3. Reis, B. F., Zagatto, E. A. G., Jacintho, A. O., Krug, F. J. and Bergamin F°., H., *Anal. Chim. Acta*, **119** (1980), 305.
4. Araujo, A. N., Lima, J. L. F. C., Rangel, A. S. S., Alonso, J., Batroli, J. and Barber, R., *Analyst*, **114** (1989), 1465.
5. Martelli, P. B., Reis, B. F., Kronka, E. A. M., Korn, M., Bergamin F°., H., Zagatto, E. A. G., Lima, J. L. F. C. and Araujo, A., *Anal. Chim. Acta*, **308** (1995), 397.
6. Saurina, J. and Hernandes-Cassou, S., *Analyst*, **124** (1999), 745.
7. Rios, A., Luque de Castro, M. D. and Valcárcel, M., *Anal. Chem.*, **57** (1985), 1803.
8. Santos, S. R. B., Araújo, M. G. U., Honorato, R. S., Zagatto, E. A. G., Lima, J. F. C. and Lapa, R. A. S., *Autom. Meth. & Mgmt. Chem.*, **22** (2000), 83.
9. Rocha, F. R. P. and Reis, B. F., *J. Chem. Educ.*, **77** (2000), 258.
10. Ferher, Z., Nagy, G., Slezsak, I., Toth, K. and Pungor, E., *Anal. Chim. Acta*, **273** (1993), 521.
11. Liu, H. H. and Dasgupta, P. K., *Anal. Chim. Acta*, **289** (1994), 347.
12. Trojanowicz, M. and Szponarlobinska, J., *Anal. Chim. Acta*, **230** (1990), 125.
13. Trojanowicz, M., Worsfold, P. J. and Clinch, J. R., *Trends Anal. Chem.*, **7** (1988), 301.
14. Rocha, F. R. P. and Reis, B. F., *Anal. Chim. Acta*, **409** (2000), 227.
15. Dasgupta, P. K., Bellamy, H. S., Liu, H., Lopez, J. L., Loree, E. L., Morris, K., Petersen, K. and Mir, K. A., *Talanta*, **40** (1993), 53.
16. Gaiao, E. N., Honorato, R. S., Santos, S. R. B. and Araújo, M. C. U., *Analyst*, **124** (1999), 1727.
17. Araújo, M. C. U., Santos, S. R. B., Silva, E. A., Veras G., Lima, J. L. F. C. and Lapa, R. A. S., *Quim. Nova*, **20** (1997), 137.
18. Zagatto, E. A. G., Bergamin F°., H., Brienza, S. M. B., Arruda, M. A. Z., Nogueira, A. R. A. and Lima, J. L. F. C., *Anal. Chim. Acta*, **261** (1992), 59.
19. Reis, B. F., Giné, M. F., Zagatto, E. A. G., Lima, J. L. F. C. and Lapa, R. A., *Anal. Chim. Acta*, **293** (1994), 129.
20. Kronka, E. A. M., Reis, B. F., Korn, M. and Bergamin F°., H., *Anal. Chim. Acta*, **334** (1996), 287.
21. Martelli, P. B., Reis, B. F., Korn, M. and Rufini, I. A. J., *Braz. Chem. Soc.*, **8** (1997), 479.
22. Reis, B. F., Morales-Rubio, A. and La Guardia, M., *Anal. Chim. Acta*, **392** (1999), 265.
23. Lapa, R. A. S., Lima, J. L. F. C. and Santos, J. L. M., *Anal. Chim. Acta*, **407** (2000), 225.

